# Relationship between bleeding episodes, health‐related quality of life and direct costs in adults with severe haemophilia A: Secondary analyses from the CHESS study

**DOI:** 10.1111/hae.14616

**Published:** 2022-06-30

**Authors:** Jamie O'Hara, Declan Noone, Maureen Watt

**Affiliations:** ^1^ HCD Economics Daresbury Cheshire UK; ^2^ Department of Health and Social Care University of Chester Chester UK; ^3^ Takeda Pharmaceuticals International AG Zurich Switzerland

**Keywords:** Cost of illness, haemophilia A, healthcare economics, quality of life, surveys and questionnaires

To the Editors,

1

The prevalence of significant morbidities and the high cost of care associated with severe haemophilia A contribute to both impaired health‐related quality of life (HRQoL) for patients and significant financial impact on healthcare budgets.^1^, ^2^, ^3^ Findings from CHESS (Cost of Haemophilia across Europe—a Socioeconomic Survey), a questionnaire‐based cost‐of‐illness survey in patients with haemophilia A and B in five European countries (France, Germany, Italy, Spain and the United Kingdom), revealed the total annual cost of severe haemophilia in 2014 was €1.55 billion (just under €200,000 per patient), driven by consumption of clotting factor replacement therapy.^4^ Estimated total indirect costs, driven by patient and caregiver work loss, were €43.3 million (€6075 per patient). However, the relationship between the number of bleeding episodes experienced by patients and their HRQoL as well as the impact on direct costs were not explored in depth. In this secondary analysis, we evaluated data from CHESS to increase our understanding of the impact of bleeding on HRQoL and on drug‐related and non‐drug‐related direct costs among adults with severe haemophilia A without inhibitors.

The full methodology of the retrospective cross‐sectional CHESS study has been published previously.^4^ Briefly, 996 consenting male patients, ≥18 years of age, with severe haemophilia A (factor VIII level < 1%) were included in the study.^4^ Individuals with inhibitors, acquired haemophilia or other clotting factor deficiencies were excluded.^4^ Data were collected between December 2014 and April 2015, and included a period of 12 months of retrospective follow‐up.^4^ Data were collected by means of two questionnaires, designed specifically for clinicians or patients.^4^ The patient questionnaire included the utility index of the EuroQol 5‐dimension 3‐level instrument (EQ‐5D‐3L), with a higher summary index score indicating better HRQoL. Bleeding episodes were categorised as: (1) minor bleeds—mild pain, minimal joint swelling, minimal restriction of motion and resolution within 24 hours of treatment or (2) major bleeds—moderate to severe pain, effusion, limitation of motion and failure to improve within 24 hours of treatment. Patients’ current and previous treatment regimens were categorised as: primary on‐demand, primary prophylaxis, secondary on‐demand (after initial prophylactic treatment) or secondary prophylaxis (after initial on‐demand). For on‐demand regimens, factor consumption for the most recent 3‐month period was annualised. For prophylaxis regimens, mean IU per infusion were multiplied by the weekly infusion rate and annualised. For this analysis, patients were also categorised by their annualised bleeding rate (ABR) (0, 1, 2–3 or >3). Drug‐related direct unit costs for each country were calculated using publicly available cost data, as described previously.^4^ The average factor unit cost (based on 2015 costs) used in CHESS for haemophilia A was €.61 (France €.72, Germany €1.44, Italy €.83, Spain €.47 and the UK €.43). Non‐drug‐related direct costs included consultant and specialist healthcare provider visits, hospitalisations, tests and procedures, bleed‐related hospitalisations, surgical procedures related to target joints (e.g., arthroscopy, arthrodesis, arthroplasty, arthrocentesis and synovectomy) and caregiver provision. Publicly available cost data were used to develop a unit cost database for each country.

Of the original 996 patients with severe haemophilia A enrolled in CHESS,^4^ a subset of 947 patients had available clinical and cost‐of‐illness data for this secondary analysis. Of these, 255 patients received primary on‐demand treatment, 384 received secondary on‐demand treatment, 172 received primary prophylaxis and 136 received secondary prophylaxis. Of the 947 patients, 404 (43%) completed the EQ‐5D‐3L questionnaire and were included in the HRQoL analysis. Our findings revealed an inverse relationship between mean EQ‐5D‐3L utility index scores and ABR category, indicating worse HRQoL in patients with high bleed rates (Figure 1). This relationship was stronger for the pain/discomfort and anxiety/depression domains than for the mobility, self‐care and usual activities domains. The relationship between lower numbers of bleeding episodes and higher mean EQ‐5D‐3L scores was more evident among patients experiencing major bleeds than minor bleeds. However, the ABR had a greater impact on utility index score than bleed severity (Figure 1), indicating that every bleed is significant to the patient. This suggests that new approaches may be required to assess the cumulative impact of bleeds in patients with haemophilia over time, whereby even a single minor bleed could have a deleterious effect on a patient's HRQoL.

**FIGURE 1:**
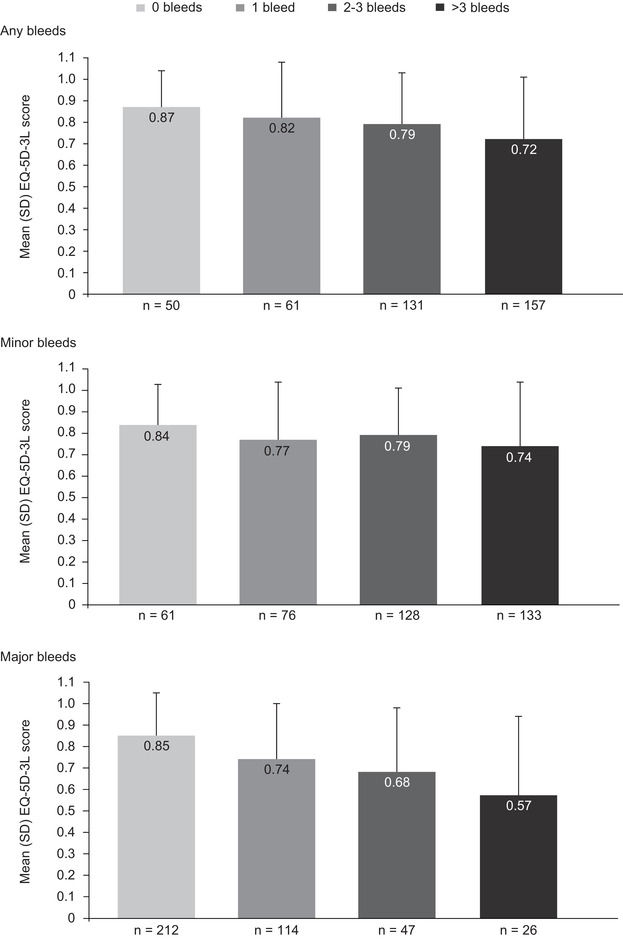
EuroQol 5‐dimension 3‐level (EQ‐5D‐3L) utility index scores by ABR category. ABR, annualised bleeding rate; SD, standard deviation

We evaluated drug‐related and non‐drug‐related direct costs associated with bleeding episodes in the 947 patients with severe haemophilia A. In the evaluable population, mean (standard deviation) annual per‐patient drug‐related and non‐drug‐related direct costs associated with any bleeding episodes were €192,913 (€181,578) and €8253 (€13,810), respectively, compared with €129,536 (€127,310) and €3909 (€7853), respectively, for patients with no recorded bleeding episodes. Non‐drug‐related annual costs reached €11,215 for patients with >3 bleeding episodes; however, there was high variation among patients with an ABR of >3, which could be explained by the small number of respondents in this group. As a result, under‐representation of patients with high rates of bleeding may have undervalued the true impact of such high bleeding rates. Non‐drug‐related direct costs were driven by hospitalisations, specialist visits and tests, because patients with frequent bleeding episodes, both major and minor, reported a high utilisation of medical resources, particularly for scheduled haemophilia consultations, nurse visits and physiotherapy.

Generalised linear regression models were utilised to determine the impact of each additional recorded major and minor bleed on direct costs. For every year a patient aged, non‐drug‐related direct costs increased by an average marginal effect (AME) of €74 (major bleed) and €78 (minor bleed; *P* < .01; Table 1). The number of target joints was also associated with increased non‐drug‐related direct costs, with the AME ranging from €1832 to €2533 (Table 1). After controlling for these variables, the additional impact of each recorded bleed per year had an AME of €2489 (*P *< .01) for a major bleed and €145 (not significant) for a minor bleed (Table 1). These findings suggest that the impact on the health system of non‐drug‐related costs remains constant per recorded major bleed, without a cumulative effect.

**TABLE 1:** Regression analysis of bleed variables for non‐drug‐related and drug‐related direct costs

	Non‐drug‐related direct costs		Drug‐related direct costs	
	Annual major bleed	Annual minor bleed	Annual major bleed	Annual minor bleed
Annual cost, €	2489*** ± 671	145 ± 114	8092* ± 4001	11,944*** ± 1554
Per 1‐year increase in age, €	74*** ± 26	78*** ± 25	−349** ± 138	205* ± 113
Cost by treatment strategy, €				
Secondary prophylaxis	−93 ± 775	949 ± 817	198,972*** ± 16,830	202,803*** ± 15,705
Prophylaxis	−478 ± 510	−687 ± 502	173,739*** ± 21,622	188,475*** ± 20,165
Secondary on‐demand	520 ± 1158	2071* ± 1239	25,625*** ± 8855	33,260*** ± 7419
Cost by number of target joints, €	1832*** ± 27	2533*** ± 461	6629* ± 3992	1283 ± 2176
Cost by history of inhibitors, €				
Once	75,571*** ± 25,908	26,562 ± 18,912	38 ± 1531	431 ± 1645
More than once	155,716* ± 82,296	79,014 ± 72,966	10,125 ± 7809	11,191 ± 8011
Constant	50,341*** ± 7848	4503 ± 4707	582 ± 741	459 ± 763

* *P* < .1;

** *P* < .05;

*** *P* < .01.

For drug‐related direct costs, generalised linear regression models found the impact of each additional recorded bleed, after controlling for variables, to have an AME of €8092 (*P* < .1) for major bleeds and €11,944 (*P* < .01) for minor bleeds. Drug costs associated with cumulative bleeding episodes were non‐linear, indicating that greater treatment consumption is necessary to gain control of bleeding episodes. Evidence supporting this assertion was found in the treatment strategy variable, where both drug‐related and non‐drug‐related direct costs were consistently higher for secondary prophylaxis. However, these findings may over‐simplify the impact of additional bleeds on drug costs, because some patients on prophylaxis may adjust their treatment schedule rather than require additional factor concentrates, with a minimal net effect on annual usage. Nonetheless, the additional costs associated with secondary prophylaxis in patients who have target joints and history of an inhibitor underscore the need to promote timely primary prophylactic replacement therapy.^5^

This analysis has several limitations. These include the uncertainty often associated with the clinical diagnosis and definition of bleeds, meaning that several minor bleeds could have had a non‐haemophilia pathophysiology. In addition, the fact that patients with severe arthropathy and stiff joints sometimes do not experience bleeds may have impacted the results. Although, our analysis attempted to create a transparent model for the main factors influencing EQ‐5D‐3L, some factors were not considered, such as body mass index, human immunodeficiency virus and hepatitis C virus seropositivity, and sociodemographic factors.^6^, ^7^, ^8^, ^9^ Further, patients experiencing few bleeds may have adapted to their condition, resulting in minimal impact on daily living scores observed on HRQoL assessment.^10^ Patient adherence to treatment may also have impacted results. As this is a retrospective analysis of data from medical records and patient questionnaires, errors in data transfer or recall cannot be ruled out and not all bleeds may have been reported in the patient records. In addition, patient numbers at the individual time points in each analysis were different, which may have exacerbated selection bias.

Although, treatment paradigms have changed in recent years, with new therapies becoming available, real‐world evidence from studies such as CHESS still provides important insights into the treatment and management of patients with rare diseases such as haemophilia. Bleeding episodes still contribute to significant morbidity in patients with severe disease and have a profound impact on QoL. This analysis further highlights the impact of the occurrence and severity of bleeding episodes on both drug‐related and non‐drug‐related direct costs and on HRQoL. Few previous studies have used a simultaneous, standardised methodology across multiple countries and combined multiple levels of burden for severe disease.

In conclusion, our findings reveal that the occurrence and severity of bleeding episodes in patients with severe haemophilia A have profound effects on HRQoL and both drug‐related and non‐drug‐related direct costs, with even a single bleeding episode having a measurable impact. Substantial costs beyond the cost of prophylaxis were observed. Prevention of any bleeding episode is an important consideration for clinicians and patients when managing severe haemophilia A, in terms of quality of life and healthcare resource allocation.

## CONFLICTS OF INTEREST

J.O. is an employee of HCD Economics. D.N. is a consultant for HCD Economics. M.W. is an employee of Takeda Pharmaceuticals International AG, and a Takeda stock owner.

## AUTHOR CONTRIBUTIONS

All authors contributed to the study concept and design, participated in reviewing and interpreting the data and the manuscript, and have read and approved the final manuscript before submission.

2

## Data Availability

The data that support the findings of this study are available from the corresponding author upon reasonable request.

## References

[1] Chen SL . Economic costs of hemophilia and the impact of prophylactic treatment on patient management. Am J Manag Care. 2016;22(5 Suppl):s126‐s133. 27266809

[2] Guh S , Grosse SD , McAlister S , Kessler CM , Soucie JM . Healthcare expenditures for males with haemophilia and employer‐sponsored insurance in the United States, 2008. Haemophilia. 2012;18(2):268‐275. 22151000 10.1111/j.1365-2516.2011.02692.x PMC4530317

[3] Guh S , Grosse SD , Mcalister S , Kessler CM , Soucie JM . Health care expenditures for medicaid‐covered males with haemophilia in the United States, 2008. Haemophilia. 2012;18(2):276‐283. 22188641 10.1111/j.1365-2516.2011.02713.x PMC4684173

[4] O'hara J , Hughes D , Camp C , Burke T , Carroll L , Garcia Diego D‐A . The cost of severe haemophilia in Europe: the CHESS study. Orphanet J Rare Dis. 2017;12(1):106. 28569181 10.1186/s13023-017-0660-y PMC5452407

[5] Srivastava A , Santagostino E , Dougall A , et al. WFH guidelines for the management of Hemophilia, 3rd edition. Haemophilia. 2020;26(Suppl 6):1‐158. 32744769 10.1111/hae.14046

[6] Royal S , Schramm W , Berntorp E , et al. Quality‐of‐life differences between prophylactic and on‐demand factor replacement therapy in European haemophilia patients. Haemophilia. 2002;8(1):44‐50. 11886464 10.1046/j.1365-2516.2002.00581.x

[7] Siboni SM , Mannucci PM , Gringeri A , et al. Health status and quality of life of elderly persons with severe hemophilia born before the advent of modern replacement therapy. J Thromb Haemost. 2009;7(5):780‐786. 19220727 10.1111/j.1538-7836.2009.03318.x

[8] Walsh M , Macgregor D , Stuckless S , Barrett B , Kawaja M , Scully M‐F . Health‐related quality of life in a cohort of adult patients with mild hemophilia A. J Thromb Haemost. 2008;6(5):755‐761. 18284605 10.1111/j.1538-7836.2008.02929.x

[9] Fischer K , Van Der Bom JG , Van Den Berg HM . Health‐related quality of life as outcome parameter in haemophilia treatment. Haemophilia. 2003;9(Suppl 1):75‐81. discussion 82. 12709041 10.1046/j.1365-2516.9.s1.13.x

[10] Dolan P , Kahneman D . Interpretations of utility and their implications for the valuation of health. Econ J. 2008;118(525):215‐234.

